# Combined transcriptome and metabolome analysis of chicken follicles in Tengchong Snow Chicken follicle selection

**DOI:** 10.5713/ab.24.0861

**Published:** 2025-04-11

**Authors:** Yanli Du, Xiannian Zi, Kun Wang, Jinshan Ran, Hanyi Xiang, Li Lei, Yong Liu

**Affiliations:** 1 College of Agronomy and Life Sciences, Kunming University, Kunming, China; 2 College of Animal Science and Technology, Yunnan Agricultural University, Kunming, China; 3 Yunnan Rural Revitalizing Education Institute, Yunnan Open University, Kunming, China; 4 Key Laboratory of Buffalo Genetics, Breeding and Reproduction Technology, Guangxi Bufialo Research Institute, Chinese Academy of Agricultural Sciences, Nanning, China; 5 Luxi Kangtai Fruit and Animal Husbandry Production and Marketing Co., Luxi, China

**Keywords:** Egg-laying Chickens, Follicle, Metabolome Analysis, Reverse Transcription Quantitative Polymerase Chain Reaction, Transcriptome Analysis

## Abstract

**Objective:**

The development of pre-hierarchical follicles (PHFs), especially small yellow follicles (SYFs), directly affects the recruitment of dominant follicles and subsequently affects the egg-laying performance of chickens. The development of PHFs, especially SYFs, and their regulatory mechanisms remain unclear.

**Methods:**

Transcriptomic and metabolomic analyses were conducted on large white follicles (LWFs) and SYFs in chickens.

**Results:**

Transcriptome sequencing revealed 258 differentially expressed genes (DEGs) between SYFs and LWFs, with 172 genes upregulated and 86 downregulated. The DEGs were mapped to 17 Kyoto encyclopedia of genes and genomes (KEGG) pathways, including glutathione metabolism, ferroptosis, calcium signaling pathway, and neuroactive ligand–receptor interactions, among others. The metabolome analysis revealed 129 significant differential metabolites (DMs), comprising 36 upregulated and 93 downregulated metabolites. The DMs were associated with nine KEGG pathways, including glutathione metabolism, alpha-linolenic acid metabolism, and linoleic acid metabolism, etc. The combined transcriptional and metabolic analysis revealed significantly enriched pathways. Five KEGG pathways associated with follicular development were identified, including glutathione metabolism, ferroptosis, alpha-linolenic acid metabolism, linoleic acid metabolism, and pyrimidine metabolism.

**Conclusion:**

Glutathione metabolism may directly inhibit Ferroptosis, which can induce apoptosis of granulosa cells, thereby regulating SYFs development in chickens. These findings could serve as a reference for improving the egg-laying performance of Tengchong snow chickens.

## INTRODUCTION

Egg-laying performance is a critial economic indicator in the chicken breeding process and has garnered significant attention from researchers. The Tengchong snow chicken, an indigenous breed exclusive to Baoshan City, Yunnan Province, China, is renowned for its superior dark meat quality, including its skin, muscle, and internal organs [1]. However, this breed exhibits relatively low egg production. Consequently, enhancing its egg-laying capacity has become a priority for both industrial development and genetic resource conservation.

Egg-laying performance depends on the development and selection of ovarian follicles in chickens [2]. This is an extremely complex process that eventually results in atresia or develops until ovulation [3]. Follicles in chickens start developing with the recruitment of the original follicle, which continues to grow, undergoes selection, and finally matures and ovulates [4]. After hens become sexually mature, their ovaries are filled with many follicles at different stages of development, usually arranged according to a strict hierarchy and generally divided into the pre-hierarchical follicle (PHF) and hierarchical follicles (HFs) stages [5]. According to the volume (from small to large) and yolk color, the former can be divided into the small white follicle (diameter, 1 to 4 mm) stage, the large white follicle (LWFs; diameter, 4 to 6 mm) stage, and the small yellow follicle (SYFs; diameter, 6 to 8 mm) stage. The latter is divided into the F5 to F1 stages according to volume, from small to large; follicles in the F1 stage are about to ovulate, followed by the F2 stage, and so on. Each stage is a tightly controlled process [6]. The follicle reserve at each stage may affect the ovulation cycle and, ultimately, ovulation efficiency, especially in the SYF stage [7]. To maintain sustained ovulation, 1 to 2 SYFs are selected from the HF stage every day to enter the rank follicle stage, and they then start growing rapidly and eventually differentiate to become the dominant follicles, a process also known as follicle selection [8]. The greater the number of 6 to 8 mm SYFs selected to enter the HFs stage, the longer the laying cycle and the better the egg-laying performance. Therefore, the development of PHFs, especially SYFs, directly impacts dominant follicle recruitment, necessitating a thorough investigation of PHFs development and their regulatory mechanisms to improve egg-laying performance.

In this study, we focused on the LWF and SYF stages, critical phases of follicle selection that represent follicles before and after selection in chickens. We conducted a combined transcriptomic and metabolomic analysis to identify the regulatory genes, metabolites, and signaling pathways involved in follicle development and selection in chickens. Our findings provide new insights into the morphological changes and molecular mechanisms underlying follicle selection.

## MATERIALS AND METHODS

### Animals and sample collection

All animal experimental procedures were approved by the Kunming University Animal Care and Use Committee (approval ID: kmu2024042). Tengchong snow chickens (n = 24) obtained from Baoshan, Yunnan Province, China, were raised at the experimental chicken farm of Kunming University under identical environmental conditions with a 10 h/14 h light/dark cycle. At 26 weeks of age, when egg-laying performance peaks, the hens were euthanized via intravenous injection of 0.5 mL of 10% potassium chloride solution, and their follicles were collected. Samples for RNA sequencing and real-time quantitative polymerase chain reaction (RT-qPCR) were immediately frozen in liquid nitrogen and then stored at −80°C.

### Histological observation of follicles

At the age of 26 weeks, 24 hens were slaughtered, and their ovaries were collected and dissected. Follicles were fixed in 4% (vol/vol) buffered formaldehyde for 24 h, dehydrated in a graded ethanol series, and embedded in paraffin. Sections were stained with hematoxylin and eosin and observed under a microscope.

### Transcriptome analysis

#### RNA extraction, cDNA library construction, and sequencing

Total RNA was extracted from LWFs and SYFs (n = 4) using TRIzol reagent (Invitrogen, Carlsbad, CA, USA) according to the manufacturer’s instructions. Specifically, Trizol reagent was used to lyse all cells in the LWFs and SYFs, release intracellular RNA, denaturate proteins, egg yolk and RNase, and thereby protecting RNA from degradation. RNA quality control was performed using an Agilent 2100 bioanalyzer (Agilent Technologies, Santa Clara, CA, USA) to detect the integrity and total volume of RNA. A cDNA library was constructed from the high-quality RNA samples. High-throughput sequencing was conducted on the Illumina HiSeq 5000 platform of Beijing Novo Technology Co., Ltd. (Beijing, CN); using a paired-end 150 bp sequencing strategy. The sequencer captured fluorescence signals and converted them into sequence data using specialized software, thereby obtaining the sequence information of the tested fragments. Eight biological replicates were used for RNA-seq analysis.

#### Bioinformatics analyses

The sequence fragments were converted into sequence data (reads) using CASAVA (version 1.8) base recognition. The RNA-seq data were preprocessed with fastq (version 0.19.7) to remove adapters and low-quality sequences. Clean reads were aligned to the chicken reference genome (ftp://ftp.ncbi.nlm.nih.gov/genomes/all/GCF_000002315.4_Gallus_gallus-5.0_genomic.gff.gz) using Hisat 2 software (version 2.0.5). The resulting SAM files were converted to BAM format using SAMtools (version 1.5). We visualized the bam files using the Integrative Genomics Viewer for quality assessment. Gene expression quantification was performed using the featureCounts tool from the subread package (version 2.0.1). Cufflinks (version 2.2.1) was used to normalize gene expression levels based on the number of fragments per kilobase of transcript per million mapped reads. HTSeq (version 0.6.1) was employed to count the number of reads mapping to each gene. Differentially expressed genes (DEGs) were identified using DESeq2 (version 1.32.0), with screening criteria set at |log2 fold change (FC)| ≥ 1 and padj < 0.05. The DEGs from all comparison groups were compiled into a comprehensive differential gene set. Gene ontology functional enrichment analysis and KEGG pathway enrichment analysis were conducted using clusterProfiler.

#### Quantitative reverse transcription polymerase chain reaction

To determine the accuracy and repeatability of the RNA-seq results, eight genes were selected for RT-qPCR to measure their expression levels in different follicles. Primers for the amplification of candidate genes were designed using the Prime-Blast tools from the NCBI database, including NPC intracellular cholesterol transporter 2, glutaredoxin domain-containing cysteine-rich protein 2 (GRXCR2), arginine vasopressin receptor 1B (AVPR1B), bone morphogenetic protein 15 (BMP15), doublesex and mab-3 related transcription factor 2 (DMRT2), stimulated by retinoic acid gene 6 (STRA6), glutathione synthetase (GSS), glutathione peroxidase 4 (GPX4), and glycoprotein IX platelet (GP9). These primers are listed in Supplement 1.

Total RNA was extracted from LWFs and SYFs (n = 6) using a method identical to the one used for transcriptome analysis, and cDNA was synthesized using a reverse transcription kit (TaKaRa, Dalian, China). RT-qPCR was performed on a CFX96TM Real-Time System (Bio-Rad, Hercules, CA, USA) using a SYBR premix Ex TaqTM II kit (Tiangen, Beijing, China). The 2-ΔΔCT method was used to determine gene expression levels, and beta-actin was used as the endogenous reference gene for normalization.

### Metabolomics analysis

#### Metabolite extraction

Metabolites were extracted from chicken SYFs and LWFs tissues. Four replicates were used for each group, and eight libraries were constructed. Metabolites were extracted using 80% methanol as the organic solvent and dissolved via ultrasonic treatment. The metabolites and organic solvents were separated by centrifugation. Detailed procedures are as follows: 100 mg of tissue samples were homogenized in liquid nitrogen and transferred to an EP tube, followed by the addition of 500 μL of 80% methanol aqueous solution. The mixture was incubated in an ice bath for 5 minutes and then centrifuged at 15,000×g at 4°C for 20 minutes. A certain amount of the supernatant was collected and diluted with ultrapure water to reduce the methanol content to 53%. The diluted mixture was subjected to another centrifugation at 15,000×g at 4°C for 20 minutes, and the final supernatant was collected for liquid chromatography-tandem mass spectrometry analysis.

#### Liquid chromatography-tandem mass spectrometry conditions

The metabolites were separated using a Vanquish UHPLC (Thermo Fisher, Waltham, MA, USA) Hypesil Gold column (Thermo Fisher). The column temperature was 40°C, and the flow rate was 0.2 mL/min. The mobile phase consisted of buffer A (0.1% formic acid) and buffer B (methanol). Throughout the analysis, the samples (2 μL) were placed in an automatic injector at 4°C. Quality control samples were inserted into the sample queue to monitor and evaluate the stability of the system and the reliability of the test data. The samples were analyzed with a high-resolution mass spectrometer (Q Exactive HF-X; Thermo Fisher) in both positive and negative electrospray ionization ion modes.

#### Metabolomics data analysis

The KEGG database (https://www.genome.jp/kegg/pathway.html), HMDB (https://hmdb.ca/metabolites), and LIPIDMaps database (http://www.lipidmaps.org/) were used to identify the metabolites.

For multivariate statistical analysis, the metabolomics data processing software metaX was used to transform the data, and then principal component analysis (PCA) and partial least square discriminant analysis (PLS-DA) were performed to obtain the variable importance in projection (VIP) value of each metabolite. In the univariate analysis, the statistical significance (p-value) of each metabolite between the two groups was calculated by conducting t-tests, and the FC of the metabolite between the two groups was calculated. The default criteria for differential metabolite screening were VIP>1, p<0.05, and FC≥2 or FC≤0.5. A volcano map was drawn using the R package (version 3.4.3) ggplot 2, and the three parameters of the VIP value, log2 (FC), and −log10 (p-value) of the metabolites were integrated to screen the metabolites of interest.

A bubble map was drawn using ggplot2, and the KEGG database was used to study the functions and metabolic pathways of the metabolites. When x/n>y/n, the metabolic pathway was considered enriched. The metabolic pathway was considered significantly enriched when the p-value was <0.05.

### Conjoint analysis of the metabolome and transcriptome

The metabolome and transcriptome were analyzed together. Common significant signaling pathways between the transcriptome and metabolome were identified and displayed using the online Wien tool (https://www.omicstudio.cn/tool). To investigate the correlation between DEGs and DMs, a combined analysis of the selected DEGs and DMs from both the metabolome and transcriptome was performed, followed by constructing a transcriptional-metabolite network with a Pearson correlation coefficient exceeding 0.7 (https://www.omicstudio.cn/tool).

### Statistical analyses

Follicular histological differences between LWFs and SYFs were determined using the Student’s t-test in SPSS 22.0 (SPSS Inc, Chicago, USA). The data were presented as the mean ±standard deviation. All differences among and between groups were considered statistically significant at p<0.05; one asterisk (*) indicates p<0.05, and two asterisks (**) indicate p<0.01.

## RESUILS

### Histological observation of follicles

**Figure 1:**
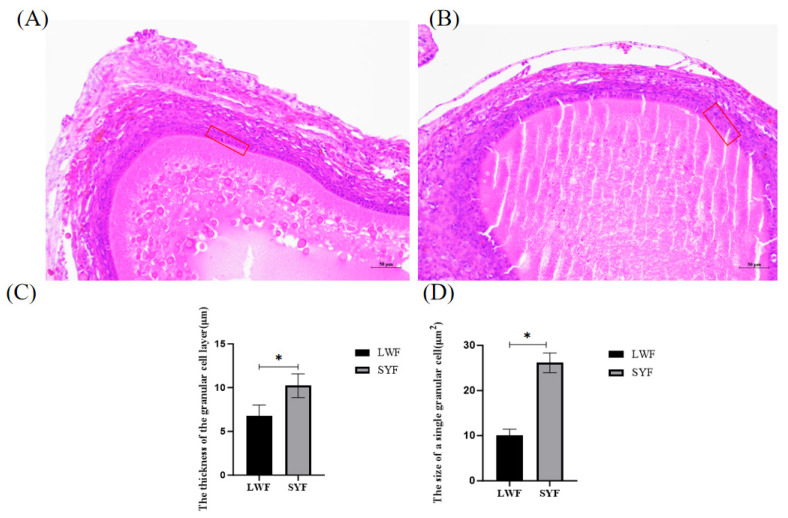
Follicular histological differences between large white follicles (LWFs) and small yellow follicles (SYFs). Histological characteristics of LWF (A) and SYF (B) samples. (C) Thickness of the granulosa cell (GC) layer. (D) The area of a single GC. H&E staining at 200×. The red rectangles indicate the GC layer; scale bar = 50 μm. * p<0.05.

One or two layers of granulosa cells (GCs) were found in the LWFs (Figure 1A), and two layers of GCs were found in the SYFs (Figure 1B). During follicle development, the SYFs GC layers (9.92±1.20 μm; Figures 1B, 1C) were significantly thicker than those of the LWF GCs (7.92±1.30 μm; Figures 1A, 1C). The area of single GCs in the SYFs was significantly greater than in the LWFs, and the GCs in the LWFs were more densely arranged (Figures 1A, 1B, and 1D).

### Transcriptome analysis

#### Data summary of the transcriptome

Eight cDNA libraries were constructed using the samples obtained from the SYFs and LWFs groups. In total, 379.34 million raw reads were obtained. After adaptors and low-quality reads were removed, 374.79 million clean reads were obtained. The average number of clean reads was 97.92% for Q20 and 94.21% for Q30. The average GC content of the clean reads was 49.05%. Eight cDNA library sequences were mapped to the genome, and the comparison range was 88.25–90.52% (Supplement 2). FASTQ files were submitted to the NCBI database. The BioProject ID is PRJNA1179835.

### Analysis of differentially expressed genes

**Figure 2:**
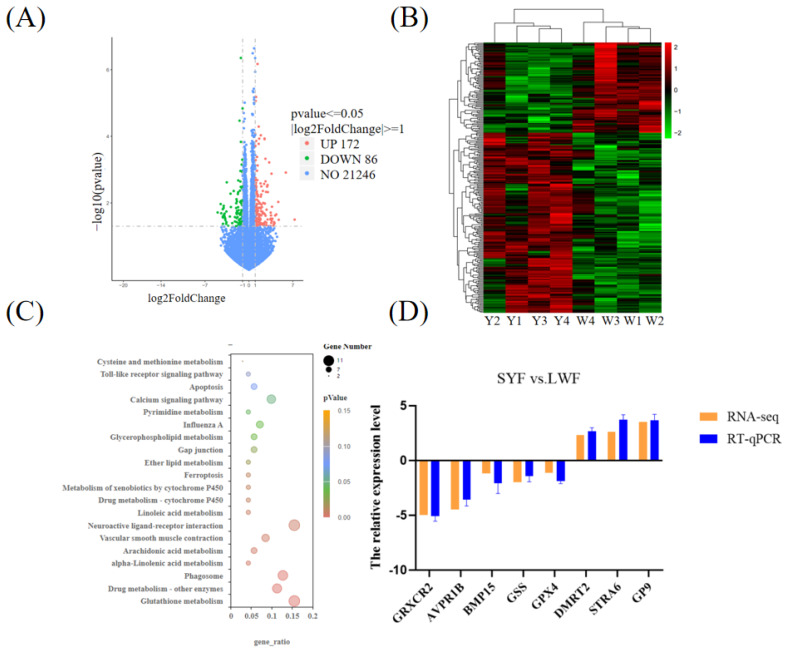
Transcriptome analysis of SYFs vs. LWFs. (A) DEGs in SYFs and LWFs. (B) Hierarchical cluster analysis of DEGs. Red indicates upregulation, and green indicates downregulation. (C) KEGG analysis of DEGs in SYFs and LWFs. (D) Validation of DEGs via RT-qPCR in SYFs and LWFs. Y represents the SYFs, and W represents the LWFs. SYF, small yellow follicle; LWF, large white follicles; RT-PCR, real-time polymerase chain reaction; GRXCR2, glutaredoxin domain-containing cysteine-rich protein 2; AVPR1B, arginine vasopressin receptor 1B; BMP15, bone morphogenetic protein 15; GSS, glutathione synthetase; GPX4, glutathione peroxidase 4; DMRT2, doublesex and mab-3 related transcription factor 2; STRA6, stimulated by retinoic acid gene 6; GP9, glycoprotein IX platelet; DEG, differentially expressed genes; KEGG, Kyoto encyclopedia of genes and genomes.

A total of 258 DEGs were identified in SYFs vs. LWFs, of which 172 were upregulated and 86 were downregulated (Figure 2A; Supplement 3). In the heat map, the color differences reflect the DEG information. The samples from the same group were clustered together to visualize the gene expression patterns of each group (Figure 2B).

### Kyoto encyclopedia of genes and genome analysis of differentially expressed genes

The DEGs were functionally enriched using KEGG. We obtained 17 KEGG signaling pathways in SYFs vs. LWFs, including Glutathione metabolism, Ferroptosis, Drug metabolism—other enzymes, Phagosome, alpha-Linolenic acid metabolism, Arachidonic acid metabolism, Vascular smooth muscle contraction, Neuroactive ligand–receptor interaction, Linoleic acid metabolism, Drug metabolism—cytochrome P450, Metabolism of xenobiotics by cytochrome P450, Ether lipid metabolism, Gap junction, Glycerophospholipid metabolism, Influenza A, Pyrimidine metabolism, and the Calcium signaling pathway (Figure 2C; Supplement 4).

#### Verification of differentially expressed genes using real-time quantitative polymerase chain reaction

We randomly selected DEGs (GRXCR2, AVPR1B, BMP15, DMRT2, STRA6, GSS, GPX4, and GP9) for RT-qPCR validation. The results showed that the relative expression levels of three genes (DMRT2, STRA6, and GP9) were greater in SYFs than in LWFs. The relative expression levels of Five genes (GRXCR2, AVPR1B, GSS, GPX4, and BMP15) were lower in SYFs than in LWFs (Figure 2D). The relative mRNA levels of the selected genes were consistent with the results of the transcriptome analysis.

### Metabolomics analysis

#### Analysis of principal component analysis and orthogonal partial least square discriminant analysis

**Figure 3:**
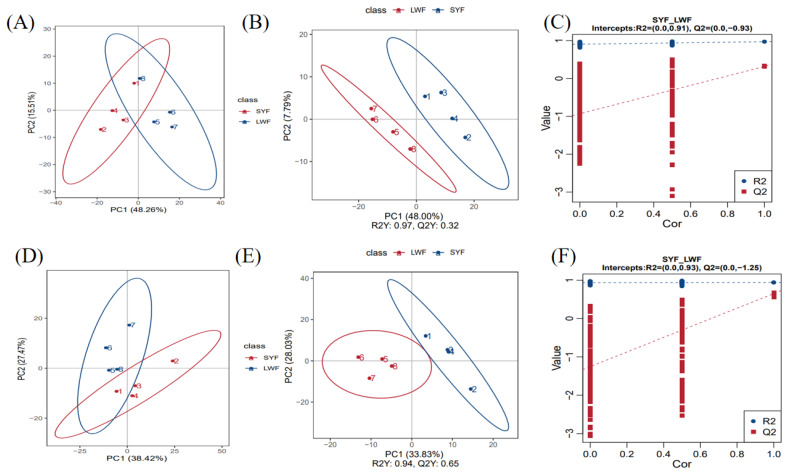
Metabolome quality control analysis of SYFs and LWFs. (A and D) PCA for the negative and positive models. (B and E) OPLS-DA for the negative and positive models. (C and F) Permutation test of OPLS-DA for the negative and positive models. PC, principal component; SYF, small yellow follicle; LWF, large white follicles; PCA, principal component analysis; OPLS-DA, orthogonal partial least square discriminant analysis.

PCA is an unsupervised multivariate statistical analysis method used to determine overall metabolic differences between groups and changes in samples within each group. The PCA score plot results showed that the overall metabolite distribution trend in the SYFs and LWFs samples was separated in the negative model (Figure 3A) and the positive model (Figure 3D). Additionally, by plotting the orthogonal partial least square discriminant analysis score, a clear separation of metabolites between the SYFs and LWFs groups was found in the negative model (Figure 3B) and the positive model (Figure 3E). Displacement tests were conducted to determine the accuracy of the OPLS-DA model (Figures 3C, 3F). In conclusion, PCA and OPLS-DA models of the follicle samples from the SYFs and LWFs groups in positive and negative ion modes were stable and reliable, indicating that the samples were reproducible enough to be used for subsequent analysis.

#### Analysis of DMdifferential metabolites

**Figure 4:**
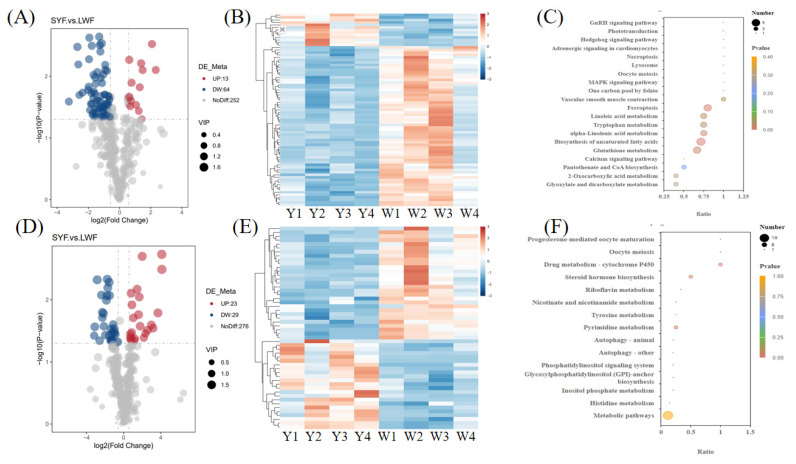
Metabolomic analysis of SYFs vs. LWFs. The DMs are shown in the negative (A) and positive (D) models. Hierarchical cluster analysis of DMs in the negative (B) and positive (E) models. Red indicates upregulation, and blue indicates downregulation. KEGG analysis of DMs in the negative (C) and positive (F) models. Y represents the SYFs, and W represents the LWFs. VIP, variable importance in projection; SYF, small yellow follicle; LWF, large white follicles; DM, differential metabolite; KEGG, Kyoto encyclopedia of genes and genomes.

A total of 129 significant DMs were identified in SYFs vs. LWFs, of which 36 were upregulated and 93 were downregulated. The 129 significant DMs comprised 77 significant DMs (including 13 upregulated and 64 downregulated DMs) in the negative model (Figure 4A; Supplement 5) and 52 significant DMs (including 23 upregulated and 29 downregulated DMs) in the positive model (Figure 4D; Supplement 6). In the heat map, the color differences reflect the DM information. The samples from the same group clustered together, and the heat map visually reflected the metabolomic expression patterns of the three groups (Figures 4B, 4E).

#### Kyoto encyclopedia of genes and genomes analysis of differential metabolites

The DMs in the SYFs and LWFs groups were functionally enriched using KEGG. In total, nine significantly enriched pathways were identified, with seven in the negative model (Figure 4C; Supplement 7) and two in the positive model (Figure 4F; Supplement 8). The pathways included Biosynthesis of unsaturated fatty acids, Ferroptosis, Glutathione metabolism, alpha-Linolenic acid metabolism, Tryptophan metabolism, Linoleic acid metabolism, Vascular smooth muscle contraction, Drug metabolism—cytochrome P450, and Pyrimidine metabolism (Figures 4C, 4F; Supplements 7, 8).

### Integrated analysis of transcriptomics and metabolomics

**Figure 5:**
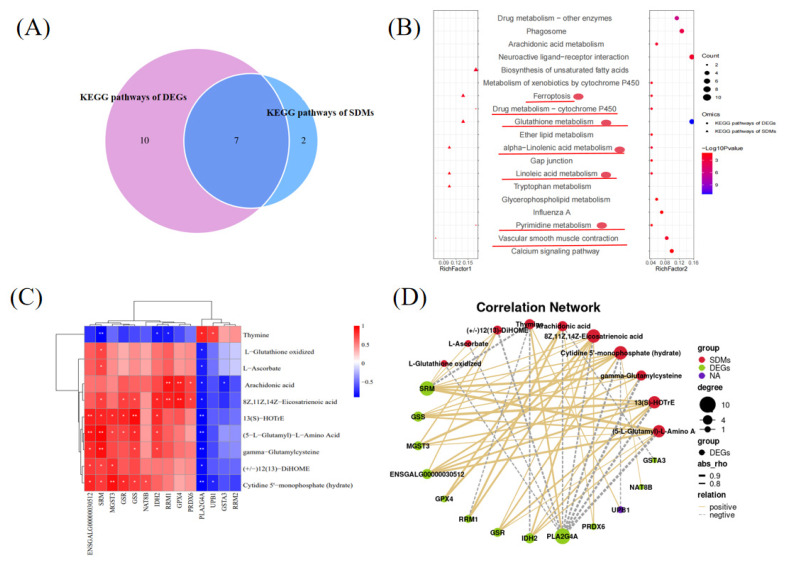
Conjoint analysis of metabolomic and transcriptomic data. (A) Venn diagram of KEGG pathways shared between the transcriptome and metabolome. (B) Shared pathway analysis of the transcriptomics and metabolomics data. (C) Correlation heat map of the metabolomic and transcriptomic data for the integrated pathway. (D) Correlation network of DEGs and DMs for the shared pathway. * p<0.05, ** p<0.01. KEGG, Kyoto encyclopedia of genes and genomes; SDM, significantly different metabolite; DEG, differentially expressed gene; DM, differential metabolite.

The KEGG analysis of the DEGs and DMs showed that the metabolome and transcriptome shared seven KEGG pathways, namely, Glutathione metabolism, Ferroptosis, alpha-Linolenic acid metabolism, Linoleic acid metabolism, Pyrimidine metabolism, Drug metabolism—cytochrome P450, and Vascular smooth muscle contraction (Figures 5A, 5B). This study focused on five KEGG pathways associated with follicular development, including Glutathione metabolism, Ferroptosis, alpha-Linolenic acid metabolism, Linoleic acid metabolism, and Pyrimidine metabolism (Figure 5B). We examined the correlations between DMs and DEGs related to Glutathione metabolism, Ferroptosis, alpha-Linolenic acid metabolism, Linoleic acid metabolism, and Pyrimidine metabolism. A Pearson correlation analysis of 10 metabolites and 14 genes showed that 13 genes were significantly positively or negatively correlated with 1 or more of the 10 metabolites (Figures 5C, 5D).

**Figure 6:**
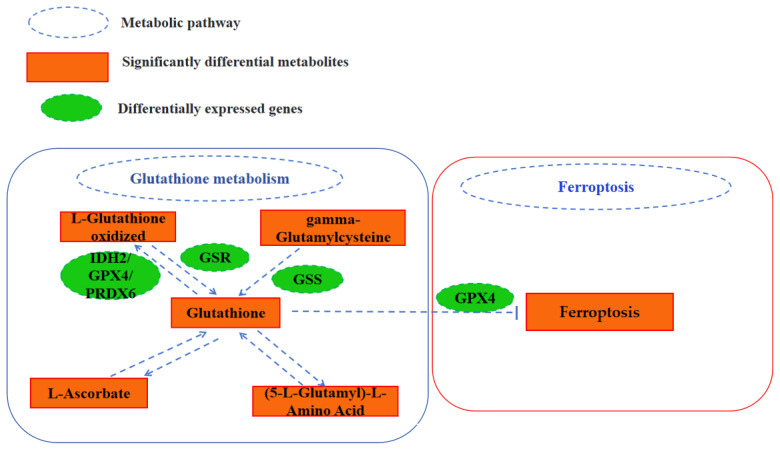
Analysis of key metabolism pathway, metabolites and genes that may regulate follicular development in SYFs vs. LWFs. SYF, small yellow follicle; LWF, large white follicles.

Based on the above results and the KEGG pathway database, we identified a network that may regulate follicular development in Tengchong snow chickens (Figure 6), mainly involving pathways ranging from glutathione metabolism to ferroptosis associated with significantly different metabolites and DEGs. However, the regulatory network of follicular development in chickens is very complex, and only a small part of this network is understood. Therefore, further studies are needed to investigate and confirm these findings.

## DISCUSSION

To enhance egg-laying production performance, it is crucial to study the SYFs development and their regulatory mechanism, as this directly affects the recruitment of dominant follicles. GCs, a key component of ovarian follicles, undergo rapid proliferation during follicular development to supply nutrients to these follicles [9]. Subsequently, GCs then proliferate rapidly and differentiate into grade GCs that can secrete various factors to regulate the maturation of oocytes and steroid hormone production [10]. We prepared paraffin sections of chicken follicles and observed their morphological characteristics. Our findings revealed that the GC layer in SYFs was significantly thicker than that in LWFs during follicle selection. This increased thickness results from GC proliferation, facilitating the transition from pre-layered follicles to layered follicles [11]. As the GC layer develops, the area of individual GCs increases, and the arrangement becomes more loosely organized. Therefore, understanding SYFs development and its regulatory mechanisms is essential for improving egg-laying performance. However, the specific molecular regulatory mechanisms involved require further elucidation.

Our KEGG pathway analysis revealed that the DEGs identified in the SYFs and LWFs groups were associated with 17 significant signaling pathways, including Ferroptosis, Glutathione metabolism, Calcium signaling pathway, and Neuroactive ligand–receptor interactions, etc. Ferroptosis is a new type of programmed cell death that accumulates lipid reactive oxygen species, leading to cell death [12]. Studies have shown that dehydroepiandrosterone treatment increases the expression of transferrin receptor 1 and iron content in mouse ovaries, promoting Ferroptosis of GCs, and subsequent GCs death [13]. Overexpression of miR-93-5p has also been demonstrated to induce GC death through Ferroptosis [14]. The Ferroptosis pathway may play a role in the transition from LWFs to SYFs and in maintaining SYFs reserves in chickens, thereby initiating the biological process of follicle selection [15]. Therefore, Ferroptosis may directly influence GCs proliferation or apoptosis during the LWFs-to-SYFs transition stage, affecting follicular development during this critical period in Tengchong Snow chickens. This pathway involves the GSS and GPX4 genes. GSS can mediate oocyte maturation in starfish by acting on the ovary to produce the maturation-inducing hormone, 1-methyladenine, which, in turn, induces the maturation of oocytes [16]. Study has shown that Ferroptosis is mainly related to glutathione depletion and GPX4 inactivation [17]. Glutathione depletion inactivates GPX4 and damages the ferroptosis defensive system, further promoting Ferroptosis [18]. Interestingly, Glutathione metabolism was significantly enriched in our study. This pathway also involves the GSS and GPX4 genes. Therefore, Glutathione metabolism may affect GCs proliferation and apoptosis during the LWFs-to-SYFs transition stage through Ferroptosis, thus affect LWFs and SYFs development in chickens. The Calcium signaling pathway that initiates development in many mammalian follicles has been extensively studied [19]. It involves the glutamate ionotropic receptor NMDA type subunit 2A (GRIN2A), epidermal growth factor (EGF), and fibroblast growth factor 8 genes. GRIN2A mediates estrogen [20], which regulates GCs proliferation and can be used as a biomarker to predict oocyte maturation in GCs. EGF directly affects development of isolated goat secondary follicles [21]. Neuroactive ligand–receptor interaction is closely related to SYFs development, and egg production in poultry [22]. This pathway involves the P2Y receptor family member 8 (P2RY8), purinergic receptor P2X2 (P2RX2), and GRIN2A genes. No study has reported the relationship between the P2RY8, P2RX2, and GRIN2A genes and follicle development, necessitating further investigation. Thus, these significantly enriched signaling pathways may affect follicle development during the LWFs-to-SYFs transition stage in chickens.

In the present study, our KEGG pathway analysis revealed that the DMs identified in the SYFs and LWFs groups were associated with nine significant signaling pathways, including Glutathione metabolism, alpha-Linolenic acid metabolism, Linoleic acid metabolism, and Pyrimidine metabolism, etc. Glutathione helps protect mature ovarian follicles from apoptotic stimuli [23]. Additionally, glutathione deficiency affects germ cell apoptosis in cultured embryonic mouse ovaries [24]. These findings suggest that Glutathione metabolism may be involved in follicle development during LWFs-to-SYFs transition stage in Tenchong snow chickens. Alpha-Linolenic acid metabolism significantly affects oocyte development in cattle [25]. Pyrimidine metabolism is closely related to ovarian cells development in Chinese hamsters [26], and Linoleic acid metabolism induces human ovarian GCs inflammation and apoptosis through the ER-FOXO1-ROS-NFκB pathway [27]. These results suggest these significantly enriched signaling pathways may affect follicle development during the LWFs-to-SYFs transition stage in Tenchong snow chickens.

The Venn diagram plotted in this study indicated that the metabolome and transcriptome shared seven pathways. Of these, five may be associated with follicular development, including Glutathione metabolism, Ferroptosis, alpha-Linolenic acid metabolism, Linoleic acid metabolism, and Pyrimidine metabolism. Correlation analysis of DMs and DEGs in these five pathways showed that most genes were hightly correlated with metabolites (Figures 5C, 5D). Among them, we focused on the GSS, and GPX4 genes. It has been discussed above that GSS and GPX4 are closely related to function of ovary [16–18]. In this study, GSS was significantly positively correlated with Cytidine 5’-monophosphate (hydrate), gamma-Glutamylcysteine, (5-L-Glutamyl)-L-Amino Acid, 13(S)-HOTrE, and 8Z,11Z,14Z-Eicosatrienoic acid, and no significant negatively correlation with DMs. GPX4 was significantly positively correlated with 8Z,11Z,14Z-Eicosatrienoic acid and Arachidonic acid, and no significant negatively correlation with DMs. gamma-Glutamylcysteine is a precursor of glutathione, which is used by GSS to form glutathione [28]. 13(S)-HOTrE is associated with ovarian dysfunction [29]. Arachidonic acid facilitates ovaries development at developmental stage II in Chinese sturgeon [30]. At present, the relationship between (5-L-Glutamyl)-L-Amino Acid and ovarian follicles has not been reported, and further research is needed. The same is true of 8Z,11Z,14Z-Eicosatrienoic acid. Therefore, it was hypothesized that GSS and GPX4 may be important genes in regulating the function of ovarian follicles in chickens.

Our findings showed that Glutathione metabolism and Ferroptosis play a important role in regulating follicle development during LWFs-to-SYFs transition stage. In addition, three other pathways may also be closely related to follicle development during LWFs-to-SYFs transition stage, including alpha-linolenic acid metabolism, linoleic acid metabolism, and pyrimidine metabolism. However, these three signal pathways were not significantly associated with Glutathione metabolism or ferroptosis in the KEGG database. Through comprehensive analysis, we found a regulatory network for chicken oocyte development during LWFs-to-SYFs transition stage, Glutathione metabolism, and Ferroptosis, including key DEGs and DMs. In figure 6, Glutathione metabolism directly inhibits the Ferroptosis pathway. The Ferroptosis can induce apoptosis of GCs [13,14], thereby affecting follicular development during LWFs-to-SYFs transition stage in chickens. Therefore, Glutathione metabolism plays an vital role in increasing egg production.

## CONCLUSION

To summarize, we analyzed the genetic landscape of ovarian follicle development and selection in chickens. We identified five signaling pathways associated with follicular development by combining transcriptomic and metabolomic data. We also found that glutathione metabolism may affect ovarian follicle development during LWFs-to-SYFs transition stage through Ferroptosis to regulate the SYFs reserve process in chickens. However, the genetic mechanisms underlying follicle development during LWFs-to-SYFs transition stage are very complex, and the functions and regulatory networks associated with these important metabolites and genes need to be further studied. The results will help us understand the molecular processes of follicle development and may provide a theoretical basis for studying the molecular regulatory mechanisms underlying chicken follicle development during LWFs-tos-SYF transition stage.

## SUPPLEMENTARY MATERIAL

Supplementary files is from: https://doi.org/10.5713/ab.24.0861

**Supplement 1**. Primers for RT-qPCR **Supplement 2**. RNA-sequencing reads and mapping rate

**Supplement 3**. DEGs between SYF and LWF

**Supplement 4**. KEGG of DEGs between SYF and LWF

**Supplement 5**. DMs between SYF and LWF in the negative model

**Supplement 6**. DMs between SYF and LWF in the positive model

**Supplement 7**. KEGG of DMs in the negative model

**Supplement 8**. KEGG of DMs in the positive model
