# Supplementary material for: Combined transcriptome and metabolome analysis of chicken follicles in Tengchong Snow Chicken follicle selection
Source: Anim Biosci. 2025 Apr 11;38(7):1316–27. doi: 10.5713/ab.24.0861 (PMC12229924; doi:10.5713/ab.24.0861)
Supplement: Supplementary file 1 [file ab-24-0861-Supplementary-1,2.pdf]

Supplement 1. Primers for RT-qPCR

| Gene | Accession number | Primers (5'->3') | Product length (bp) |
|---|---|---|---|
| GRXCR2-F | NM_001416574.1 | ACTGCTATGGCAGCAGAAGG | 108 |
| GRXCR2-R | | TAAAGCCCAGAGAAGGCACC | |
| AVPR1B-F | NM_001031498.2 | CCGTGCAGGGAGATGTAAGG | 127 |
| AVPR1B-R | | CAGGTTGTCACCTCTGTCCC | |
| BMP15-F | NM_001006589.3 | CCGGGACCTCTTTCTGCTTT | 97 |
| BMP15-R | | GTGGATGACAAGGGGTGAGG | |
| DMRT2-F | XM_046936552 | AGACGGGCTTTTGCCGATAA | 173 |
| DMRT2-R | | GCATCAACCTGAGAAGGGCT | |
| STRA6-F | NM_001293202 | CTCAACAACCCCTCGCTGAT | 251 |
| STRA6-R | | CCTTGGCTGTGTCATCACCT | |
| GP9-F | NM_001389561 | TTCCCCTTCAGAGTCCCAGT | 136 |
| GP9-R | | TGGGGTACATTCAGTGGCTG | |
| GSS-F | XM_040688003.2 | TTGGGACAGGAACATTCGGG | 94 |
| GSS-R | | GCCATCCACATAGAGCCTCC | |
| GPX4-F | NM_204220.3 | TCCATCTACGACTTCCACGC | 116 |
| GPX4-R | | ACCGCGGTCTTTCCTCATTT | |
| ACTB-F | NM_205518.1 | ACCGGACTGTTACCAACACC | 116 |
| ACTB-R | | CCTGAGTCAAGCGCCAAAAG | |

Supplement 2. RNA-sequencing reads and mapping rate

| Sample | Raw Data | | Clean Data | | Q20% | Q30% | GC% | Total_map (%) |
|---|---|---|---|---|---|---|---|---|
| | Reads | Base (G) | Reads | Base (G) | | | | |
| SYF 1 | 47,622,830 | 7.14 G | 46,967,682 | 7.05 G | 97.88 | 94.17 | 49.4 | 41,684,424 (88.75%) |
| SYF 2 | 47,146,628 | 7.07 G | 46,589,606 | 6.99 G | 97.93 | 94.2 | 48.35 | 41,113,933 (88.25%) |
| SYF 3 | 47,446,224 | 7.12 G | 46,985,756 | 7.05 G | 97.94 | 94.17 | 47.21 | 41,887,553 (89.15%) |
| SYF 4 | 48,006,310 | 7.2 G | 47,432,074 | 7.11 G | 98.03 | 94.49 | 49.32 | 42,688,934 (90.0%) |
| LWF1 | 47,178,292 | 7.08G | 46,654,706 | 7.0 G | 97.95 | 94.26 | 48.29 | 42,233,384 (90.52%) |
| LWF2 | 49,372,418 | 7.41G | 48,738,738 | 7.31 G | 97.9 | 94.2 | 50.23 | 43,987,272 (90.25%) |
| LWF3 | 48,580,864 | 7.29 G | 47,975,658 | 7.2 G | 97.86 | 94.1 | 49.53 | 43,168,732 (89.98%) |
| LWF4 | 43,986,970 | 6.6 G | 43,449,124 | 6.52 G | 97.83 | 94.05 | 50.03 | 38,870,397 (89.46%) |

Supplement 3. DEGs between SYF and LWF

| gene_id | log2FoldChange | pvalue | gene_name |
|---|---|---|---|
| ENSGALG00000011347 | -1.277771319 | 4.44E-07 | IHH |
| ENSGALG00000017235 | 1.368690365 | 6.74E-07 | NOX4 |
| ENSGALG00000015008 | 1.054421058 | 6.47E-06 | ZNF366 |
| ENSGALG00000016959 | 1.132984998 | 6.58E-06 | DGKH |
| ENSGALG00000014537 | 1.072357636 | 8.65E-06 | BMF |
| ENSGALG00000014513 | -1.012503759 | 1.46E-05 | CDCA3 |
| novel.248 | -1.495552411 | 3.39E-05 | - |
| ENSGALG00000028304 | 1.554453193 | 5.10E-05 | MMR1L4 |
| ENSGALG00000011076 | 1.001054325 | 8.51E-05 | SYNE3 |
