# Supplementary material for: Combined transcriptome and metabolome analysis of chicken follicles in Tengchong Snow Chicken follicle selection
Source: Anim Biosci. 2025 Apr 11;38(7):1316–27. doi: 10.5713/ab.24.0861 (PMC12229924; doi:10.5713/ab.24.0861)
Supplement: Supplementary file 2 [file ab-24-0861-Supplementary-3.pdf]

Supplement 1. Primers for RT-qPCR

Gene	Accession number	Primers (5'->3')	Product length (bp)
GRXCR2-F	NM_001416574.1	ACTGCTATGGCAGCAGAAGG	108
GRXCR2-R		TAAAGCCCAGAGAAGGCACC	
AVPR1B-F	NM_001031498.2	CCGTGCAGGGAGATGTAAGG	127
AVPR1B-R		CAGGTTGTCACCTCTGTCCC	
BMP15-F	NM_001006589.3	CCGGGACCTCTTTCTGCTTT	97
BMP15-R		GTGGATGACAAGGGGTGAGG	
DMRT2-F	XM_046936552	AGACGGGCTTTTGCCGATAA	173
DMRT2-R		GCATCAACCTGAGAAGGGCT	
STRA6-F	NM_001293202	CTCAACAACCCCTCGCTGAT	251
STRA6-R		CCTTGGCTGTGTCATCACCT	
GP9-F	NM_001389561	TTCCCCTTCAGAGTCCCAGT	136
GP9-R		TGGGGTACATTCAGTGGCTG	
GSS-F	XM_040688003.2	TTGGGACAGGAACATTCGGG	94
GSS-R		GCCATCCACATAGAGCCTCC	
GPX4-F	NM_204220.3	TCCATCTACGACTTCCACGC	116
GPX4-R		ACCGCGGTCTTTCCTCATTT	
ACTB-F	NM_205518.1	ACCGGACTGTTACCAACACC	116
ACTB-R		CCTGAGTCAAGCGCCAAAAG	

Supplement 2. RNA-sequencing reads and mapping rate

Sample	Raw Data		Clean Data		Q20%	Q30%	GC%	Total_map (%)
	Reads	Base (G)	Reads	Base (G)				
SYF 1	47,622,830	7.14 G	46,967,682	7.05 G	97.88	94.17	49.4	41,684,424 (88.75%)
SYF 2	47,146,628	7.07 G	46,589,606	6.99 G	97.93	94.2	48.35	41,113,933 (88.25%)
SYF 3	47,446,224	7.12 G	46,985,756	7.05 G	97.94	94.17	47.21	41,887,553 (89.15%)
SYF 4	48,006,310	7.2 G	47,432,074	7.11 G	98.03	94.49	49.32	42,688,934 (90.0%)
LWF1	47,178,292	7.08G	46,654,706	7.0 G	97.95	94.26	48.29	42,233,384 (90.52%)
LWF2	49,372,418	7.41G	48,738,738	7.31 G	97.9	94.2	50.23	43,987,272 (90.25%)
LWF3	48,580,864	7.29 G	47,975,658	7.2 G	97.86	94.1	49.53	43,168,732 (89.98%)
LWF4	43,986,970	6.6 G	43,449,124	6.52 G	97.83	94.05	50.03	38,870,397 (89.46%)

Supplement 3. DEGs between SYF and LWF

gene_id	log2FoldChange	pvalue	gene_name
ENSGALG00000011347	-1.277771319	4.44E-07	IHH
ENSGALG00000017235	1.368690365	6.74E-07	NOX4
ENSGALG00000015008	1.054421058	6.47E-06	ZNF366
ENSGALG00000016959	1.132984998	6.58E-06	DGKH
ENSGALG00000014537	1.072357636	8.65E-06	BMF
ENSGALG00000014513	-1.012503759	1.46E-05	CDCA3
novel.248	-1.495552411	3.39E-05	-
ENSGALG00000028304	1.554453193	5.10E-05	MMR1L4
ENSGALG00000011076	1.001054325	8.51E-05	SYNE3

ENSGALG00000053491	1.887468658	9.32E-05	ENSGALG00000053491
ENSGALG00000043254	2.485005602	0.000116272	EPX
ENSGALG00000009239	1.634921566	0.000117406	TLR2B
ENSGALG00000024272	2.488871709	0.000121839	S100A12
ENSGALG00000014699	1.158761726	0.000137492	MEGF10
ENSGALG00000030512	-1.279290101	0.000146846	ENSGALG00000030512
ENSGALG00000043064	1.549526579	0.000167944	EXFABP
ENSGALG00000037387	1.032332428	0.000195073	CLSTN2
ENSGALG00000002786	1.282343535	0.000416612	PSTPIP1
ENSGALG00000001449	2.629562654	0.000472582	STRA6
novel.844	-1.068010829	0.000504138	-
ENSGALG00000030065	1.076044522	0.000517564	TENM3
ENSGALG00000034294	3.229472902	0.000607601	ATP6V0D2
ENSGALG00000046656	-1.153663849	0.000706015	PMVK
ENSGALG00000025881	1.385036372	0.000748565	ENSGALG00000025881
ENSGALG00000043415	1.279364037	0.001077783	ACP3
ENSGALG00000044406	-1.35153942	0.001094695	ENSGALG00000044406
ENSGALG00000040260	-1.071370006	0.00116823	TUBA1C
ENSGALG00000014989	5.87286469	0.001222243	S100Z
ENSGALG00000016325	3.597418598	0.001321968	GSTA3
ENSGALG00000016687	1.047035855	0.001781595	P2RY8
ENSGALG00000005065	1.335511101	0.002052867	PLA2G4A
ENSGALG00000003137	1.07174884	0.002066154	RFLNA
ENSGALG00000039489	-1.706497853	0.002107825	5_8S_rRNA
ENSGALG00000051701	2.642475904	0.002247268	ENSGALG00000051701
novel.755	-1.359967621	0.002275883	-
ENSGALG00000027702	-3.527782152	0.002410947	SCN11A
ENSGALG00000050893	1.12469757	0.002478682	CCR2
ENSGALG00000053205	1.841961679	0.002585623	ENSGALG00000053205
ENSGALG00000034946	2.419657554	0.002688815	ENSGALG00000034946
ENSGALG00000002080	1.0171119	0.002772312	DOCK2
ENSGALG00000014144	1.016335261	0.002816474	CORIN
ENSGALG00000019553	2.967925386	0.002891232	SERPINB10B
ENSGALG00000008442	1.073128372	0.00294707	TENM1
ENSGALG00000007337	1.020905595	0.002963475	LPP
ENSGALG00000016919	1.130541241	0.003148676	ACOD1
ENSGALG00000051018	1.972114868	0.003189182	ENSGALG00000051018
novel.447	-1.033237587	0.003218996	-
ENSGALG00000049622	1.154420776	0.003221466	ENSGALG00000049622
ENSGALG00000043582	2.892344255	0.003233522	ENSGALG00000043582
ENSGALG00000003039	-1.295238891	0.00328874	SRM
ENSGALG00000031518	-1.059097568	0.003405563	ENSGALG00000031518
ENSGALG00000016227	1.023911113	0.00345295	GPR34
ENSGALG00000010461	1.142632576	0.003467986	EBF3

ENSGALG00000054787	1.099882232	0.003477709	ENSGALG00000054787
ENSGALG00000003445	-1.128796172	0.003629023	MGST3
ENSGALG00000051897	1.102083348	0.003910264	ENSGALG00000051897
ENSGALG00000015307	1.104963147	0.003987772	ENSGALG00000015307
ENSGALG00000044649	-1.431776089	0.004263143	P2RX2
ENSGALG00000053499	2.21812145	0.004360567	ENSGALG00000053499
ENSGALG00000036082	-2.42484733	0.004368497	HOXC13
ENSGALG00000030195	1.249785534	0.004503991	ENSGALG00000030195
ENSGALG00000010943	2.733804147	0.00466197	ENSGALG00000010943
ENSGALG00000049091	3.014479806	0.004722805	ENSGALG00000049091
ENSGALG00000011349	-1.71569224	0.004768413	ENSGALG00000011349
ENSGALG00000040276	1.237984962	0.004988803	RUNX2
ENSGALG00000005472	1.227356261	0.005201784	NAT
ENSGALG00000007278	1.006282548	0.005206705	GRIN2A
novel.675	-2.933578426	0.00527325	-
ENSGALG00000048550	1.244677105	0.005295967	ENSGALG00000048550
ENSGALG00000017350	-1.765764424	0.005395197	RRM1
ENSGALG00000016693	1.124942994	0.005398802	CSF2RA
ENSGALG00000040557	1.961686046	0.005572342	TFEC
ENSGALG00000009315	1.432787127	0.005757335	PAPLN
ENSGALG00000016590	1.450976063	0.006215406	TLR7
ENSGALG00000049636	1.356069586	0.006412597	ENSGALG00000049636
ENSGALG00000016442	1.879476718	0.006464217	RRM2
ENSGALG00000003281	-1.98612622	0.007717369	GSS
ENSGALG00000026781	1.101491669	0.007920936	ALOX5AP
ENSGALG00000005985	1.298366547	0.007983061	GDF10
ENSGALG00000051984	-1.016125333	0.008179892	ENSGALG00000051984
ENSGALG00000041228	-1.293686909	0.008761194	SELENOP2
ENSGALG00000010595	1.519799962	0.008911125	GPR65
ENSGALG00000030858	-1.913851238	0.008913546	FRMD5
ENSGALG00000037194	-1.111946584	0.008933554	ENSGALG00000037194
ENSGALG00000054625	4.669510163	0.00932245	ENSGALG00000054625
ENSGALG00000003660	-1.379644663	0.009393837	FANCF
ENSGALG00000016498	2.280374681	0.009972948	MFSD2B
ENSGALG00000047247	1.723561052	0.010080055	ENSGALG00000047247
ENSGALG00000033634	-2.026321208	0.010196687	TDRD5
ENSGALG00000037781	-1.209688702	0.010357168	GSR
novel.181	2.5411134	0.010374255	-
novel.824	2.509159116	0.010402222	-
ENSGALG00000014455	1.684688859	0.010448459	LPAR5
ENSGALG00000040035	1.265446761	0.010665891	ADRB2
ENSGALG00000000788	-4.504431886	0.010769268	AVPR1B
ENSGALG00000048410	1.22277478	0.010859543	ENSGALG00000048410
ENSGALG00000049902	1.297185275	0.01093835	ENSGALG00000049902

ENSGALG00000051842	1.988535876	0.011168403	ENSGALG00000051842
ENSGALG00000048467	-1.078843049	0.011694646	SYP
ENSGALG00000048120	-4.016168859	0.012153802	ENSGALG00000048120
ENSGALG00000007001	1.110275191	0.012307489	TLR4
ENSGALG00000016261	1.176962963	0.012354864	CYBB
novel.178	3.675516826	0.013002845	-
ENSGALG00000039687	-2.875401988	0.013264565	NIPAL4
ENSGALG00000037322	2.073040464	0.013306985	HIST1H46
ENSGALG00000044235	-1.726194487	0.013359737	MAB21L2
ENSGALG00000002192	1.050979924	0.013479495	PTPRC
ENSGALG00000042168	1.240506445	0.013798335	ENSGALG00000042168
ENSGALG00000000667	1.725570908	0.013815145	EDN2
ENSGALG00000054707	-4.031973828	0.014045576	ENSGALG00000054707
ENSGALG00000014736	1.54642205	0.014166738	ENSGALG00000014736
ENSGALG00000030420	-2.039322602	0.014229924	ECEL1
ENSGALG00000050287	1.342944937	0.014275044	ENSGALG00000050287
ENSGALG00000051110	1.87215217	0.014691245	ENSGALG00000051110
ENSGALG00000042339	1.014562586	0.015134728	ENSGALG00000042339
ENSGALG00000009405	1.323122502	0.015314892	GRIA2
ENSGALG00000051766	1.273859998	0.015347194	ENSGALG00000051766
ENSGALG00000019290	-4.15821056	0.015420297	SLC6A12
ENSGALG00000027289	2.81145295	0.015919621	ENSGALG00000027289
ENSGALG00000049356	1.167339459	0.016026298	ENSGALG00000049356
ENSGALG00000004742	-1.199464846	0.016413201	BMP15
ENSGALG00000000400	-1.637269985	0.016480685	KCNC4
ENSGALG00000013086	1.153791535	0.016713921	IKZF1
ENSGALG00000051083	1.236455551	0.017001259	ENSGALG00000051083
ENSGALG00000012119	1.457093457	0.017325544	MARCO
ENSGALG00000031768	-1.902032967	0.017340887	SPO11
ENSGALG00000009948	1.074263106	0.017458174	HHIP
ENSGALG00000006627	1.952464882	0.017660517	UPB1
ENSGALG00000019147	1.216406646	0.017716266	ENSGALG00000019147
ENSGALG00000038629	-1.167273488	0.017895194	EFNA2
ENSGALG00000033857	-3.586731139	0.018090674	NAT8B
ENSGALG00000003837	1.104004007	0.018173884	ADCY7
ENSGALG00000052451	2.128396969	0.018230604	ENSGALG00000052451
ENSGALG00000027422	1.235969939	0.018396603	gga-mir-125b-1
ENSGALG00000045847	-4.983113603	0.019338025	GRXCR2
ENSGALG00000031051	2.180345512	0.019445383	ENSGALG00000031051
ENSGALG00000003053	-1.292755186	0.019492131	PRDX6
ENSGALG00000041184	-1.802440849	0.019501435	NETO1
ENSGALG00000032887	3.469208916	0.019511835	CHIR-B3
ENSGALG00000029416	1.04225798	0.019527152	TRPC6
ENSGALG00000027351	-1.995568479	0.019968726	LRRC71

ENSGALG00000026790	2.353073904	0.020300854	DMRT2
ENSGALG00000054574	-4.409245485	0.02039761	ENSGALG00000054574
ENSGALG00000009844	1.428390992	0.020808073	ACTC1
ENSGALG00000012155	-1.182736499	0.020873165	EGF
ENSGALG00000016698	-3.867094714	0.020907182	SHOX
ENSGALG00000014845	2.176197416	0.020909422	PLCXD3
ENSGALG00000011805	1.395364539	0.020942218	ENSGALG00000011805
ENSGALG00000038884	-1.5090027	0.021209299	SRL
ENSGALG00000026553	1.071076999	0.021373137	ENSGALG00000026553
ENSGALG00000034434	-1.936571125	0.021625695	IDH2
ENSGALG00000043052	1.199527642	0.021655307	CD1B
ENSGALG00000017039	2.520587264	0.021759501	STOML3
ENSGALG00000012076	1.713256601	0.022039962	TIFA
ENSGALG00000052762	4.35626247	0.022131008	ENSGALG00000052762
ENSGALG00000014984	1.002677514	0.022478536	F2RL1
novel.558	-1.400775501	0.022768602	-
ENSGALG00000002886	-3.994604591	0.022809934	NKX2-5
ENSGALG00000047489	1.239547278	0.022860334	ENSGALG00000047489
ENSGALG00000005022	3.513411757	0.023708172	GP9
ENSGALG00000009050	1.945677206	0.023812175	CAPN3
novel.587	4.184592706	0.023813883	-
ENSGALG00000025086	2.654892139	0.024186897	ENSGALG00000025086
ENSGALG00000048973	1.590864949	0.024934565	ENSGALG00000048973
ENSGALG00000036358	-1.058721923	0.025083564	LMO3
ENSGALG00000023763	-4.14414182	0.025418859	ENSGALG00000023763
ENSGALG00000054107	-3.876766362	0.025438148	ENSGALG00000054107
ENSGALG00000039978	1.011194634	0.025507028	SLC4A1
ENSGALG00000038650	-3.035161627	0.025606797	ENSGALG00000038650
ENSGALG00000053118	-1.816928761	0.026574381	ENSGALG00000053118
ENSGALG00000039479	-2.23252971	0.026663772	BPIFC
ENSGALG00000031197	1.585666201	0.027144054	ENSGALG00000031197
ENSGALG00000041015	-4.144475317	0.027389633	ENSGALG00000041015
ENSGALG00000016680	-1.950002877	0.028422125	TFAP2B
ENSGALG00000007171	1.174493616	0.028457678	ENSGALG00000007171
novel.443	-3.782495924	0.029130451	-
ENSGALG00000046904	1.796690846	0.029339032	ENSGALG00000046904
ENSGALG00000032994	-1.230513082	0.029356163	SOX17
ENSGALG00000044996	1.201399543	0.029563166	TMEM71
ENSGALG00000046759	3.469441295	0.029573109	ENSGALG00000046759
ENSGALG00000051927	3.729025066	0.02972665	ENSGALG00000051927
ENSGALG00000054601	1.1331309	0.029797271	ENSGALG00000054601
ENSGALG00000032097	1.501681416	0.030069779	TBX20
ENSGALG00000038918	3.698049627	0.030187319	ENSGALG00000038918
ENSGALG00000010672	1.672078968	0.030264476	KCNK13

ENSGALG00000009875	-1.330028701	0.030269713	HMGA2
ENSGALG00000053727	1.234442737	0.030368888	ENSGALG00000053727
ENSGALG00000052600	-1.501125653	0.03088993	ENSGALG00000052600
ENSGALG00000042116	1.365391868	0.030971555	FUT9
ENSGALG00000016968	3.433630929	0.031286563	CCDC122
ENSGALG00000014722	2.979659581	0.031443975	ENSGALG00000014722
ENSGALG00000029393	7.277148677	0.03163114	HOXC10
ENSGALG00000045173	1.839070227	0.031906461	ENSGALG00000045173
ENSGALG00000047586	4.745328705	0.032008633	ENSGALG00000047586
ENSGALG00000044392	1.626588511	0.03250472	ENSGALG00000044392
ENSGALG00000047200	-3.75616579	0.032536785	ENSGALG00000047200
ENSGALG00000034567	-1.595805536	0.032886619	POU4F3
ENSGALG00000051085	1.180545223	0.033377676	ENSGALG00000051085
ENSGALG00000041014	1.405324186	0.034045221	ENSGALG00000041014
ENSGALG00000008166	1.256086912	0.034101393	TLR15
ENSGALG00000006591	3.617463052	0.034279929	TNNI2
ENSGALG00000045115	1.066336871	0.034330094	ENSGALG00000045115
ENSGALG00000030996	1.339854716	0.034361171	ENSGALG00000030996
ENSGALG00000007706	-2.239300616	0.035944855	FGF8
ENSGALG00000048900	1.687715556	0.036088276	ENSGALG00000048900
ENSGALG00000019231	1.198032997	0.036311026	CD200R1
ENSGALG00000002595	-1.146588994	0.036405648	GPX4
novel.450	-1.372298523	0.036490867	-
ENSGALG00000014840	2.579849751	0.036689466	C6
ENSGALG00000046534	1.130280673	0.036695392	ENSGALG00000046534
ENSGALG00000000458	-1.427248262	0.036764028	TMPRSS9
ENSGALG00000040136	1.046707872	0.037337654	PSTPIP2
ENSGALG00000006216	-1.98978427	0.037423341	MYOD1
ENSGALG00000006527	1.417738339	0.037428403	CRTAM
ENSGALG00000050121	4.006583245	0.037653153	ENSGALG00000050121
ENSGALG00000039617	-3.585112121	0.037685829	ENSGALG00000039617
novel.238	-1.378332615	0.038062057	-
ENSGALG00000013218	1.035962095	0.038381239	C3AR1
novel.353	2.769987385	0.039137075	-
ENSGALG00000000994	3.560149756	0.039667628	ENSGALG00000000994
ENSGALG00000038634	1.014725382	0.039789311	KIT
ENSGALG00000016325	1.202239776	0.040234058	GSTA3
ENSGALG00000025614	-2.661082795	0.040292528	ENSGALG00000025614
ENSGALG00000042474	-1.674687641	0.040310209	FOXD2
ENSGALG00000049789	1.947366181	0.040529357	ENSGALG00000049789
ENSGALG00000048426	1.751948385	0.040577759	ENSGALG00000048426
ENSGALG00000050486	2.352302168	0.040585972	ENSGALG00000050486
ENSGALG00000047495	4.077373992	0.04075756	LRRC10
ENSGALG00000010949	1.777226248	0.040904868	GPNMB

ENSGALG00000015419	-1.339029809	0.040938974	PENK
ENSGALG00000049494	-4.12808551	0.042240805	ENSGALG00000049494
ENSGALG00000028346	-3.619223617	0.042413223	ENSGALG00000028346
ENSGALG00000013045	2.285665185	0.042806922	TUBA8B
novel.419	1.136061559	0.043053539	-
ENSGALG00000047769	2.10820244	0.043156303	ENSGALG00000047769
ENSGALG00000051508	2.421435893	0.043602695	ENSGALG00000051508
ENSGALG00000028175	3.979916469	0.043606616	GJA9
ENSGALG00000047316	1.794760908	0.043739572	ENSGALG00000047316
ENSGALG00000041204	-1.160865581	0.044188259	IGF2BP1
novel.57	2.010588569	0.044193919	-
ENSGALG00000016684	1.547212298	0.044251823	RHAG
ENSGALG00000025509	1.079899838	0.044458334	gga-mir-1465
ENSGALG00000009479	1.730493389	0.044519071	ENSGALG00000009479
ENSGALG00000054813	2.301064625	0.045534534	ENSGALG00000054813
ENSGALG00000006025	2.785562145	0.046205032	XIRP1
ENSGALG00000013625	1.215288151	0.0462165	ENSGALG00000013625
ENSGALG00000025945	1.449421566	0.046263904	AVD
ENSGALG00000053966	-1.713097569	0.04690894	ENSGALG00000053966
ENSGALG00000035619	-1.234495041	0.047147568	MTTP
ENSGALG00000006242	-1.471616697	0.047438676	PABPN1L
ENSGALG00000053681	3.888491685	0.048123081	ENSGALG00000053681
ENSGALG00000047451	-1.648599945	0.048373533	ENSGALG00000047451
ENSGALG00000054790	-3.310307872	0.048560288	ENSGALG00000054790
ENSGALG00000053151	1.23472137	0.048567044	ENSGALG00000053151
ENSGALG00000049914	3.428814182	0.049555199	ENSGALG00000049914
ENSGALG00000035565	-1.699166525	0.049566031	ZAR1
novel.599	-2.068471958	0.049730982	-
ENSGALG00000050107	3.170276542	0.049742924	ENSGALG00000050107

Supplement 4. KEGG of DEGs between SYF and LWF

KEGGID	Description	GeneRatio	BgRatio	pvalue	geneName
gga00480	Glutathione metabolism	11/71	47/5686	6.30E-12	GSTA3/SRM/MGST3/RRM1/RRM2/GSS/GSR/NAT8B/PRDX6/IDH2/GPX4
gga00983	Drug metabolism - other enzymes	8/71	61/5686	6.87E-07	EPX/GSTA3/MGST3/NAT/RRM1/RRM2/UPB1/ENSGALG00000011805
gga04145	Phagosome	9/71	178/5686	0.000329669	MMR1L4/EPX/TLR2B/ATP6V0D2/TUBA1C/TLR4/CYBB/MARCO/TUBA8B
gga00592	alpha-Linolenic acid metabolism	3/71	26/5686	0.003948338	-/ENSGALG00000030512/PLA2G4A
gga00590	Arachidonic acid metabolism	4/71	53/5686	0.004122466	-/ENSGALG00000030512/PLA2G4A/GPX4
