# Supplementary material for: Combined transcriptome and metabolome analysis of chicken follicles in Tengchong Snow Chicken follicle selection
Source: Anim Biosci. 2025 Apr 11;38(7):1316–27. doi: 10.5713/ab.24.0861 (PMC12229924; doi:10.5713/ab.24.0861)
Supplement: Supplementary file 3 [file ab-24-0861-Supplementary-4.pdf]

ENSGALG00000015419	-1.339029809	0.040938974	PENK
ENSGALG00000049494	-4.12808551	0.042240805	ENSGALG00000049494
ENSGALG00000028346	-3.619223617	0.042413223	ENSGALG00000028346
ENSGALG00000013045	2.285665185	0.042806922	TUBA8B
novel.419	1.136061559	0.043053539	-
ENSGALG00000047769	2.10820244	0.043156303	ENSGALG00000047769
ENSGALG00000051508	2.421435893	0.043602695	ENSGALG00000051508
ENSGALG00000028175	3.979916469	0.043606616	GJA9
ENSGALG00000047316	1.794760908	0.043739572	ENSGALG00000047316
ENSGALG00000041204	-1.160865581	0.044188259	IGF2BP1
novel.57	2.010588569	0.044193919	-
ENSGALG00000016684	1.547212298	0.044251823	RHAG
ENSGALG00000025509	1.079899838	0.044458334	gga-mir-1465
ENSGALG00000009479	1.730493389	0.044519071	ENSGALG00000009479
ENSGALG00000054813	2.301064625	0.045534534	ENSGALG00000054813
ENSGALG00000006025	2.785562145	0.046205032	XIRP1
ENSGALG00000013625	1.215288151	0.0462165	ENSGALG00000013625
ENSGALG00000025945	1.449421566	0.046263904	AVD
ENSGALG00000053966	-1.713097569	0.04690894	ENSGALG00000053966
ENSGALG00000035619	-1.234495041	0.047147568	MTTP
ENSGALG00000006242	-1.471616697	0.047438676	PABPN1L
ENSGALG00000053681	3.888491685	0.048123081	ENSGALG00000053681
ENSGALG00000047451	-1.648599945	0.048373533	ENSGALG00000047451
ENSGALG00000054790	-3.310307872	0.048560288	ENSGALG00000054790
ENSGALG00000053151	1.23472137	0.048567044	ENSGALG00000053151
ENSGALG00000049914	3.428814182	0.049555199	ENSGALG00000049914
ENSGALG00000035565	-1.699166525	0.049566031	ZAR1
novel.599	-2.068471958	0.049730982	-
ENSGALG00000050107	3.170276542	0.049742924	ENSGALG00000050107

Supplement 4. KEGG of DEGs between SYF and LWF

KEGGID	Description	GeneRatio	BgRatio	pvalue	geneName
gga00480	Glutathione metabolism	11/71	47/5686	6.30E-12	GSTA3/SRM/MGST3/RRM1/RRM2/GSS/GSR/NAT8B/PRDX6/IDH2/GPX4
gga00983	Drug metabolism - other enzymes	8/71	61/5686	6.87E-07	EPX/GSTA3/MGST3/NAT/RRM1/RRM2/UPB1/ENSGALG00000011805
gga04145	Phagosome	9/71	178/5686	0.000329669	MMR1L4/EPX/TLR2B/ATP6V0D2/TUBA1C/TLR4/CYBB/MARCO/TUBA8B
gga00592	alpha-Linolenic acid metabolism	3/71	26/5686	0.003948338	-/ENSGALG00000030512/PLA2G4A
gga00590	Arachidonic acid metabolism	4/71	53/5686	0.004122466	-/ENSGALG00000030512/PLA2G4A/GPX4

gga04270	Vascular smooth muscle contraction	6/71	128/5686	0.005011947	-/ENSGALG00000030512/PLA2G4A/AVPR1B/EDN2/ADCY7
gga04080	Neuroactive ligand-receptor interaction	11/71	365/5686	0.005135529	P2RY8/P2RX2/GRIN2A/ADRB2/AVPR1B/EDN2/GRIA2/F2RL1/C3AR1/PENK/ENSGALG00000047316
gga00591	Linoleic acid metabolism	3/71	32/5686	0.007140412	-/ENSGALG00000030512/PLA2G4A
gga00982	Drug metabolism - cytochrome P450	3/71	34/5686	0.008462838	GSTA3/MGST3/ENSGALG00000011805
gga00980	Metabolism of xenobiotics by cytochrome P450	3/71	35/5686	0.009174417	GSTA3/MGST3/ENSGALG00000011805
gga04216	Ferroptosis	3/71	36/5686	0.009920041	GSS/CYBB/GPX4
gga00565	Ether lipid metabolism	3/71	47/5686	0.02043987	-/ENSGALG00000030512/PLA2G4A
gga04540	Gap junction	4/71	90/5686	0.025504917	TUBA1C/ADCY7/EGF/TUBA8B
gga00564	Glycerophospholipid metabolism	4/71	100/5686	0.035693243	DGKH/-/ENSGALG00000030512/PLA2G4A
gga05164	Influenza A	5/71	149/5686	0.037728164	TLR7/TLR4/ENSGALG00000049356/ENSGALG00000007171/PABPN1L
gga00240	Pyrimidine metabolism	3/71	63/5686	0.043497748	RRM1/RRM2/UPB1
gga04020	Calcium signaling pathway	7/71	269/5686	0.0494288	P2RX2/GRIN2A/ADRB2/AVPR1B/ADCY7/EGF/FGF8

Supplement 5. DMs between SYF and LWF in the negative model

ID	Name	FC	log2FC	Pvalue	VIP	Up.Down
Com_926_neg	Ala-Leu	0.405409652	-1.302547658	0.002278815	1.397810474	down
Com_6883_neg	23-Nordeoxycholic acid	0.261613261	-1.934492423	0.002363099	1.357262478	down
Com_7141_neg	PC 16:0_18:1;O	0.447951629	-1.158585141	0.002936795	1.6879771	down
Com_1230_neg	N-Formylkynurenine	4.221987803	2.077922411	0.002999741	1.348138154	up
Com_2957_neg	LPS 20:4	0.414947169	-1.26900043	0.003122826	1.330014774	down
Com_4338_neg	LPS 16:0	0.291202799	-1.779903871	0.0031991	1.330104029	down
Com_6522_neg	5-Methylcytidine	0.15382143	-2.700671584	0.003334357	1.397638639	down
Com_4400_neg	LPS 18:2	0.426792941	-1.22839178	0.00397294	1.327880582	down
Com_514_neg	Eicosapentaenoic acid	0.257654823	-1.956488497	0.005290345	1.29909712	down
Com_367_neg	4-Hydroxyisoleucine	1.539415739	0.622382903	0.005398894	1.341876208	up
Com_2982_neg	Pantetheine	2.609719017	1.383894483	0.006147811	1.346089407	up
Com_7192_neg	15-OxoEDE	0.160317169	-2.640999156	0.006422642	1.298803331	down
Com_256_neg	Adenosine diphosphate (ADP)	0.528259136	-0.920682282	0.006516799	1.277596729	down
Com_10258_neg	Adenosine 5'-diphosphoglucose	0.529187328	-0.918149581	0.007884227	1.320881123	down
