# Supplementary material for: Combined transcriptome and metabolome analysis of chicken follicles in Tengchong Snow Chicken follicle selection
Source: Anim Biosci. 2025 Apr 11;38(7):1316–27. doi: 10.5713/ab.24.0861 (PMC12229924; doi:10.5713/ab.24.0861)
Supplement: Supplementary file 4 [file ab-24-0861-Supplementary-5.pdf]

gga04270	Vascular smooth muscle contraction	6/71	128/5686	0.005011947	-/ENSGALG00000030512/PLA2G4A/AVPR1B/EDN2/ADCY7
gga04080	Neuroactive ligand-receptor interaction	11/71	365/5686	0.005135529	P2RY8/P2RX2/GRIN2A/ADRB2/AVPR1B/EDN2/GRIA2/F2RL1/C3AR1/PENK/ENSGALG00000047316
gga00591	Linoleic acid metabolism	3/71	32/5686	0.007140412	-/ENSGALG00000030512/PLA2G4A
gga00982	Drug metabolism - cytochrome P450	3/71	34/5686	0.008462838	GSTA3/MGST3/ENSGALG00000011805
gga00980	Metabolism of xenobiotics by cytochrome P450	3/71	35/5686	0.009174417	GSTA3/MGST3/ENSGALG00000011805
gga04216	Ferroptosis	3/71	36/5686	0.009920041	GSS/CYBB/GPX4
gga00565	Ether lipid metabolism	3/71	47/5686	0.02043987	-/ENSGALG00000030512/PLA2G4A
gga04540	Gap junction	4/71	90/5686	0.025504917	TUBA1C/ADCY7/EGF/TUBA8B
gga00564	Glycerophospholipid metabolism	4/71	100/5686	0.035693243	DGKH/-/ENSGALG00000030512/PLA2G4A
gga05164	Influenza A	5/71	149/5686	0.037728164	TLR7/TLR4/ENSGALG00000049356/ENSGALG00000007171/PABPN1L
gga00240	Pyrimidine metabolism	3/71	63/5686	0.043497748	RRM1/RRM2/UPB1
gga04020	Calcium signaling pathway	7/71	269/5686	0.0494288	P2RX2/GRIN2A/ADRB2/AVPR1B/ADCY7/EGF/FGF8

Supplement 5. DMs between SYF and LWF in the negative model

ID	Name	FC	log2FC	Pvalue	VIP	Up.Down
Com_926_neg	Ala-Leu	0.405409652	-1.302547658	0.002278815	1.397810474	down
Com_6883_neg	23-Nordeoxycholic acid	0.261613261	-1.934492423	0.002363099	1.357262478	down
Com_7141_neg	PC 16:0_18:1;O	0.447951629	-1.158585141	0.002936795	1.6879771	down
Com_1230_neg	N-Formylkynurenine	4.221987803	2.077922411	0.002999741	1.348138154	up
Com_2957_neg	LPS 20:4	0.414947169	-1.26900043	0.003122826	1.330014774	down
Com_4338_neg	LPS 16:0	0.291202799	-1.779903871	0.0031991	1.330104029	down
Com_6522_neg	5-Methylcytidine	0.15382143	-2.700671584	0.003334357	1.397638639	down
Com_4400_neg	LPS 18:2	0.426792941	-1.22839178	0.00397294	1.327880582	down
Com_514_neg	Eicosapentaenoic acid	0.257654823	-1.956488497	0.005290345	1.29909712	down
Com_367_neg	4-Hydroxyisoleucine	1.539415739	0.622382903	0.005398894	1.341876208	up
Com_2982_neg	Pantetheine	2.609719017	1.383894483	0.006147811	1.346089407	up
Com_7192_neg	15-OxoEDE	0.160317169	-2.640999156	0.006422642	1.298803331	down
Com_256_neg	Adenosine diphosphate (ADP)	0.528259136	-0.920682282	0.006516799	1.277596729	down
Com_10258_neg	Adenosine 5'-diphosphoglucose	0.529187328	-0.918149581	0.007884227	1.320881123	down

Com_471_neg	Quinaldic acid	4.992519903	2.319768179	0.007900635	1.26400104	up
Com_8372_neg	Hex2Cer 18:0;2O	2.792369964	1.481490098	0.00792311	1.288994084	up
Com_4689_neg	13(S)-HOTrE	0.57115959	-0.808034183	0.008160685	1.274028549	down
Com_2628_neg	LPS 18:1	0.372736882	-1.423770514	0.008522566	1.288238334	down
Com_727_neg	Flavin adenine dinucleotide (FAD)	0.483426935	-1.048630236	0.009442506	1.303418276	down
Com_5896_neg	D-Fructose 1,6-bisphosphate	0.314418593	-1.66924156	0.009480919	1.269890348	down
Com_7569_neg	6-Hydroxymelatonin	0.341513501	-1.549985481	0.010429977	1.425936025	down
Com_1462_neg	11(Z),14(Z)-Eicosadieno ic Acid	0.432447366	-1.209403545	0.010658358	1.255839217	down
Com_143_neg	Folinic acid	1.706068135	0.770675264	0.01271737	1.338531741	up
Com_432_neg	Guanosine	0.459606505	-1.121528878	0.012889238	1.257302873	down
Com_7135_neg	LPS 22:4	0.343997002	-1.539532104	0.013289068	1.226471108	down
Com_1699_neg	3'-Dephosphocoenzyme A	0.254510763	-1.974201427	0.014785408	1.348253815	down
Com_3474_neg	5-Methoxyindole-3-Carb aldehyde	2.455750435	1.296163955	0.015053691	1.259228129	up
Com_155_neg	8Z,11Z,14Z-Eicosatrieno ic acid	0.248932659	-2.006172575	0.016677175	1.296657514	down
Com_265_neg	N6-Succinyl Adenosine	0.320982432	-1.639433757	0.017769123	1.259426139	down
Com_5050_neg	gamma-Glutamylcystein e	0.193824673	-2.367175864	0.018431843	1.258626792	down
Com_1644_neg	11(E)-Eicosenoic Acid	0.286021167	-1.805806176	0.018679976	1.285003028	down
Com_3002_neg	MGDG O-24:4_16:0	0.332532592	-1.588432346	0.019149882	1.197880436	down
Com_12_neg	ethyl 3-cyano-2-hydroxy-6-ph enylisonicotinate	0.512278267	-0.965000406	0.019837754	1.212238089	down
Com_107_neg	Guanosine monophosphate (GMP)	0.528285832	-0.920609377	0.019865253	1.224419295	down
Com_2067_neg	FAHFA 22:4/20:3	0.167781768	-2.575342141	0.020173428	1.187815236	down
Com_52_neg	Citric acid	0.604385121	-0.726459951	0.020703366	1.201636527	down
Com_342_neg	Docosapentaenoic acid	0.434999962	-1.20091282	0.020872347	1.238682661	down
Com_4940_neg	L-Alanyl-L-Lysine	0.627862289	-0.671479931	0.021654219	1.200967893	down
Com_9591_neg	PE O-16:0_18:2	1.54785254	0.630268036	0.021714601	1.391681251	up
Com_279_neg	FAHFA 18:1/20:3	0.310753653	-1.686156745	0.022721403	1.197888612	down
Com_56_neg	Uridine monophosphate (UMP)	0.63905783	-0.645981605	0.024024897	1.169670069	down
Com_9927_neg	Phenylacetylglutamine	0.38375293	-1.38175033	0.024853097	1.17798711	down
Com_897_neg	LPC O-18:0	1.662109025	0.733015018	0.024883377	1.224838935	up
Com_7_neg	Arachidonic acid	0.356892828	-1.486437185	0.025302795	1.204212592	down
Com_144_neg	Uric acid	0.105204721	-3.248728651	0.025693905	1.272646126	down
Com_4164_neg	Tetrahydroaldosterone	0.421669238	-1.245816317	0.026047505	1.485506506	down
Com_724_neg	cis-Aconitic acid	0.591496213	-0.757559163	0.027259667	1.167615022	down

Com_583_neg	Cytidine-5'-monophosphate	0.528197166	-0.920851535	0.027716183	1.199853694	down
Com_434_neg	FAHFA 18:2/20:4	0.278207947	-1.845764466	0.027961609	1.164959337	down
Com_6786_neg	nor-6β-Oxycodol	0.291168955	-1.780071554	0.028078534	1.164111125	down
Com_2947_neg	(5-L-Glutamyl)-L-Amino Acid	0.562605494	-0.829804454	0.028593986	1.201053186	down
Com_6311_neg	Soyasaponin I	2.090546677	1.063880256	0.029158238	1.205931968	up
Com_1379_neg	Lipoic acid	0.60739176	-0.719300759	0.029290258	1.181464831	down
Com_7504_neg	LPC 19:0	1.608304024	0.685540151	0.03079478	1.175904398	up
Com_776_neg	Glycyl-L-leucine	0.492649112	-1.021367639	0.031082124	1.16516473	down
Com_6868_neg	Adenosine 3'5'-cyclic monophosphate	0.446452347	-1.163421899	0.031424558	1.327760311	down
Com_2143_neg	N-Acetyl-L-methionine	0.477706831	-1.065802587	0.032552946	1.226723827	down
Com_4994_neg	N1-[1-(3-isopropenylphenyl)-1-methylethyl]-3-oxobutanamide	0.420561594	-1.249610988	0.032796251	1.252743653	down
Com_5826_neg	N-Oleoyl Glycine	0.37031593	-1.433171482	0.033172035	1.155618186	down
Com_3556_neg	ADBICA N-pentanoic acid metabolite	0.334648037	-1.579283542	0.033209683	1.199335445	down
Com_19_neg	Elaidic acid	0.477382456	-1.066782548	0.035068495	1.14634689	down
Com_5805_neg	Docosatrienoic Acid	0.267466269	-1.902571132	0.035348718	1.140695349	down
Com_3369_neg	H-Gly-Pro-OH	0.586793853	-0.769074337	0.035625114	1.154252654	down
Com_10191_neg	5-Hydroxyindole-3-acetic acid	2.348897748	1.231983912	0.036519541	1.177523521	up
Com_1717_neg	L-Ascorbate	0.481069137	-1.055683849	0.037771477	1.153945414	down
Com_763_neg	Guanosine 5'-diphospho-ß-L-fucose	0.356233728	-1.489103977	0.038864824	1.153567924	down
Com_76_neg	Adrenic acid	0.303206104	-1.721629297	0.039316255	1.146163235	down
Com_6324_neg	(±)10(11)-EpDPA	0.345697823	-1.532416575	0.04145983	1.214158212	down
Com_33_neg	L-Glutathione oxidized	0.593171813	-0.753478052	0.042527898	1.164796297	down
Com_311_neg	(+/-)12(13)-DiHOME	0.302321604	-1.725844017	0.042816618	1.166840163	down
Com_9281_neg	Gly-Tyr-Ala	0.538807305	-0.892158684	0.043214658	1.324653736	down
Com_443_neg	FAHFA 16:0/18:2	0.468086084	-1.095154221	0.04392988	1.097012563	down
Com_1919_neg	acetyl phospahte	0.338884451	-1.561134652	0.045055687	1.294823521	down
Com_8890_neg	N-(5-Aminopentyl)acetamide	0.65215017	-0.616723885	0.045566826	1.082661471	down
Com_3446_neg	11β-Prostaglandin E2	0.485966319	-1.041071768	0.045609631	1.0695693	down
Com_468_neg	1-(4-methylphenyl)-3-(2-pyridylthio)pyrrolidine-2,5-dione	0.447921376	-1.158682579	0.045876169	1.130667485	down
Com_102_neg	LPE 22:5	2.695659517	1.430638284	0.049304255	1.083995839	up
