# Supplementary material for: Combined transcriptome and metabolome analysis of chicken follicles in Tengchong Snow Chicken follicle selection
Source: Anim Biosci. 2025 Apr 11;38(7):1316–27. doi: 10.5713/ab.24.0861 (PMC12229924; doi:10.5713/ab.24.0861)
Supplement: Supplementary file 5 [file ab-24-0861-Supplementary-6.pdf]

Supplement 6. DMs between SYF and LWF in the positive model

ID	Name	FC	log2FC	Pvalue	VIP	Up.Down
Com_3057_pos	PI-Cer 34:0;3O	17.18267663	4.102882885	0.001854438	1.791772553	up
Com_1942_pos	PC 19:2_19:2	3.998295012	1.999384924	0.001995608	1.684519575	up
Com_2828_pos	PI-Cer 36:3;3O	17.29114127	4.11196119	0.003246782	1.799694694	up
Com_7571_pos	ethyl 2-{[3-cyano-4-(2-furyl)-6-(2-thienyl)-2-pyridinyl]oxy}acetate	0.295265164	-1.759916939	0.00467008	1.623205883	down
Com_4637_pos	S-(Methyl)Glutathione	0.139273118	-2.844011277	0.004772074	1.669192738	down
Com_532_pos	LSD-d3	0.340522063	-1.55417982	0.005228812	1.606213113	down
Com_431_pos	CAR 20:1	0.306600018	-1.705570313	0.006402255	1.564195989	down
Com_7642_pos	Lysops 22:5	2.731353618	1.449616106	0.006746179	1.558928765	up
Com_1765_pos	2-cyano-N-(3-oxo-1,3-dihydro-2-benzofuran-5-yl)benzenesulfonamide	1.75628465	0.812526689	0.007828989	1.587212809	up
Com_3834_pos	CAR 21:2	0.191588605	-2.383916337	0.008394898	1.549031011	down
Com_5098_pos	beta-Nicotinamide adenine dinucleotide phosphate	0.35112897	-1.509927062	0.008516429	1.57489324	down
Com_1619_pos	1-(2-pyridin-2-yldiazenyl)-2-naphthol	0.285443011	-1.808725355	0.008586928	1.610372819	down
Com_3202_pos	Deisopropylatrazine	3.205214586	1.680420947	0.009020024	1.541188051	up
Com_3977_pos	CAR 21:3	0.277461816	-1.849638852	0.010182362	1.58688011	down
Com_2731_pos	Virginiamycin	1.858361575	0.894031229	0.01205164	1.543105761	up
Com_4223_pos	CAR 20:5	0.203609878	-2.29612054	0.015907569	1.537318996	down
Com_6279_pos	Azaspiracid-1	12.87885555	3.686932493	0.016276973	1.672093967	up
Com_7640_pos	8-Bromoguanosine	0.222407843	-2.16872043	0.016640128	1.562717868	down
Com_4485_pos	Methyl 3-indolyacetate	3.036184489	1.602259456	0.017393138	1.606740231	up
Com_3395_pos	CAR 22:2	0.204846203	-2.287386943	0.019436511	1.453833758	down

		2.058518	1.041606	0.019533	1.470075	
Com_327_pos	Riboflavin	438	371	979	194	up
		0.456062	-1.13269	0.024981	1.422873	
Com_783_pos	1-Methylhistidine	249	7342	518	737	down
	Methyl					
	N-(5-methyl-1-phenyl-1H-pyrazol-3-yl)carb	7.898331	2.981547	0.025424	1.546537	
Com_4386_pos	amate	109	849	972	527	up
		3.427322	1.777081	0.026655	1.534286	
Com_1420_pos	1-Phenyl-3-methyl-5-pyrazolone	233	839	6	683	up
		0.229913	-2.12084	0.026721	1.492414	
Com_3630_pos	CAR 14:2	008	0005	39	86	down
	7-hydroxy-3-(4-methoxyphenyl)-4H-chrome	0.116888	-3.09679	0.027251	1.542786	
Com_1880_pos	n-4-one	241	8298	105	046	down
		9.000519	3.170008	0.028660	1.516860	
Com_2761_pos	Morphine	583	288	408	713	up
	3-(5-phenyl-1,3-oxazol-2-yl)-4-(trifluoromet	0.490764	-1.02689	0.029080	1.533574	
Com_691_pos	hyl)pyridine	403	7486	659	344	down
	methyl					
	3-(6-methylpyridin-2-yl)-2,2-diphenylpropa	0.391705	-1.35215	0.029469	1.490259	
Com_2984_pos	noate	156	9976	68	948	down
		5.945862	2.571886	0.030870	1.503598	
Com_2868_pos	Heroin-d3	25	04	732	857	up
		0.508200	-0.97652	0.032642	1.500622	
Com_352_pos	Guanine	628	9938	714	601	down
		1.827478	0.869854	0.033666	1.467569	
Com_4464_pos	Thymine	326	296	678	39	up
		0.417678	-1.25953	0.034003	1.384267	
Com_667_pos	CAR 20:2	782	4239	915	861	down
		0.483613	-1.04807	0.034433	1.470759	
Com_1638_pos	Ala-Ile	106	4753	094	484	down
		5.124567	2.357430	0.034734	1.357702	
Com_4861_pos	Progesterone	638	289	655	223	up
		1.639937	0.713640	0.035762	1.483585	
Com_2221_pos	EPK	148	523	622	965	up
		0.540777	-0.88689	0.036083	1.490066	
Com_3210_pos	2,5-Furandicarboxylic acid	965	1727	912	092	down
		0.255819	-1.96679	0.037053	1.451769	
Com_3562_pos	4-Hydroxytamoxifen	988	9103	331	685	down
	4-[4-(1-phenyl-1H-1,2,4-triazol-5-yl)-1,3-thi	0.343894	-1.53996	0.037481	1.393381	
Com_6147_pos	azol-2-yl]pyridine	25	3101	645	73	down
		0.111384	-3.16638	0.037827	1.342376	
Com_2767_pos	PI 34:2	123	4494	781	223	down
		1.733609	0.793778	0.040135	1.438649	
Com_3720_pos	DLK	098	63	691	174	up

Com_5624_pos	2-[(1-benzhydrylazetan-3-yl)thio]-N'-(4-chlorobenzoyl)acetohydrazide	4.502448079	2.17070964	0.040960739	1.33989075	up
Com_2734_pos	N-[(4-hydroxy-3-methoxyphenyl)methyl]-8-methylnonanamide	1.628919974	0.703915728	0.042130782	1.333872729	up
Com_6505_pos	10-Nitrolinoleate	2.31315238	1.209860307	0.042288269	1.363856281	up
Com_1284_pos	Cytidine 5'-monophosphate (hydrate)	0.453049917	-1.142258079	0.043808341	1.40048044	down
Com_4080_pos	Estrone	0.397170448	-1.332169813	0.044254425	1.318677948	down
Com_4380_pos	5,6-Dihydroxyindole-2-Carboxylic Acid	0.39525531	-1.339143249	0.044755153	1.407540256	down
Com_2564_pos	Lysopg 18:1	1.765383744	0.819981818	0.045071864	1.394752029	up
Com_1338_pos	2-methyl-2,3,4,5-tetrahydro-1,5-benzoxazepin-4-one	2.202642505	1.13923536	0.04544272	1.402994528	up
Com_6217_pos	Kinetin	0.379791123	-1.396721909	0.045466483	1.441075906	down
Com_1110_pos	Cysteinylglycine	0.167400055	-2.578628093	0.045480445	1.459416689	down
Com_56_pos	Acetyl-L-carnitine	0.638548154	-0.647132675	0.047891232	1.409665412	down

Supplement 7. KEGG of DMs in the negative model

MapID	MapTitle	Pvalue	x	y	n	N	MetaIDs
map01040	Biosynthesis of unsaturated fatty acids	0.005148969	5	7	29	135	8Z,11Z,14Z-Eicosatrienoic acid; Docosapentaenoic acid; Eicosapentaenoic acid; Adrenic acid; Arachidonic acid
map04216	Ferroptosis	0.00760415	4	5	29	135	L-Glutathione oxidized; gamma-Glutamylcysteine; Adrenic acid; Arachidonic acid
map00480	Glutathione metabolism	0.019334461	4	6	29	135	L-Ascorbate; (5-L-Glutamyl)-L-Amino Acid; L-Glutathione oxidized; gamma-Glutamylcysteine
map00592	alpha-Linolenic acid metabolism	0.021481481	3	4	29	135	13(S)-HOTrE
map00380	Tryptophan metabolism	0.031064772	3	4	29	135	5-Hydroxyindole-3-acetic acid; N-Formylkynurenine; 6-Hydroxymelatonin
map00591	Linoleic acid metabolism	0.031064772	3	4	29	135	8Z,11Z,14Z-Eicosatrienoic acid; (+/-)12(13)-DiHOME; Arachidonic acid
map04270	Vascular smooth muscle contraction	0.044886678	2	2	29	135	Adenosine 3'5'-cyclic monophosphate; Arachidonic acid
