# Supplementary material for: Combined transcriptome and metabolome analysis of chicken follicles in Tengchong Snow Chicken follicle selection
Source: Anim Biosci. 2025 Apr 11;38(7):1316–27. doi: 10.5713/ab.24.0861 (PMC12229924; doi:10.5713/ab.24.0861)
Supplement: Supplementary file 6 [file ab-24-0861-Supplementary-7.pdf]

Com_5624_pos	2-[(1-benzhydrylazetan-3-yl)thio]-N'-(4-chlorobenzoyl)acetohydrazide	4.502448079	2.17070964	0.040960739	1.33989075	up
Com_2734_pos	N-[(4-hydroxy-3-methoxyphenyl)methyl]-8-methylnonanamide	1.628919974	0.703915728	0.042130782	1.333872729	up
Com_6505_pos	10-Nitrolinoleate	2.31315238	1.209860307	0.042288269	1.363856281	up
Com_1284_pos	Cytidine 5'-monophosphate (hydrate)	0.453049917	-1.142258079	0.043808341	1.40048044	down
Com_4080_pos	Estrone	0.397170448	-1.332169813	0.044254425	1.318677948	down
Com_4380_pos	5,6-Dihydroxyindole-2-Carboxylic Acid	0.39525531	-1.339143249	0.044755153	1.407540256	down
Com_2564_pos	Lysopg 18:1	1.765383744	0.819981818	0.045071864	1.394752029	up
Com_1338_pos	2-methyl-2,3,4,5-tetrahydro-1,5-benzoxazepin-4-one	2.202642505	1.13923536	0.04544272	1.402994528	up
Com_6217_pos	Kinetin	0.379791123	-1.396721909	0.045466483	1.441075906	down
Com_1110_pos	Cysteinylglycine	0.167400055	-2.578628093	0.045480445	1.459416689	down
Com_56_pos	Acetyl-L-carnitine	0.638548154	-0.647132675	0.047891232	1.409665412	down

Supplement 7. KEGG of DMs in the negative model

MapID	MapTitle	Pvalue	x	y	n	N	MetaIDs
map01040	Biosynthesis of unsaturated fatty acids	0.005148969	5	7	29	135	8Z,11Z,14Z-Eicosatrienoic acid; Docosapentaenoic acid; Eicosapentaenoic acid; Adrenic acid; Arachidonic acid
map04216	Ferroptosis	0.00760415	4	5	29	135	L-Glutathione oxidized; gamma-Glutamylcysteine; Adrenic acid; Arachidonic acid
map00480	Glutathione metabolism	0.019334461	4	6	29	135	L-Ascorbate; (5-L-Glutamyl)-L-Amino Acid; L-Glutathione oxidized; gamma-Glutamylcysteine
map00592	alpha-Linolenic acid metabolism	0.021481481	3	4	29	135	13(S)-HOTrE
map00380	Tryptophan metabolism	0.031064772	3	4	29	135	5-Hydroxyindole-3-acetic acid; N-Formylkynurenine; 6-Hydroxymelatonin
map00591	Linoleic acid metabolism	0.031064772	3	4	29	135	8Z,11Z,14Z-Eicosatrienoic acid; (+/-)12(13)-DiHOME; Arachidonic acid
map04270	Vascular smooth muscle contraction	0.044886678	2	2	29	135	Adenosine 3'5'-cyclic monophosphate; Arachidonic acid
