# Supplementary material for: Combined transcriptome and metabolome analysis of chicken follicles in Tengchong Snow Chicken follicle selection
Source: Anim Biosci. 2025 Apr 11;38(7):1316–27. doi: 10.5713/ab.24.0861 (PMC12229924; doi:10.5713/ab.24.0861)
Supplement: Supplementary file 7 [file ab-24-0861-Supplementary-8.pdf]

Supplement 8. KEGG of DMs in the positive model

MapID	MapTitle	Pvalue	x	y	n	N	MetaIDs
map00982	Drug metabolism - cytochrome P450	0.01163815	2	2	12	107	Morphine; 4-Hydroxytamoxifen
map00240	Pyrimidine metabolism	0.02202723	2	8	12	107	Cytidine 5'-monophosphate (hydrate); Thymine
